# Longitudinal assessment of neuropsychological and temporal/spatial gait characteristics of elderly fallers: taking it all in stride

**DOI:** 10.3389/fnagi.2015.00034

**Published:** 2015-03-18

**Authors:** Rebecca K. MacAulay, Ted D. Allaire, Robert M. Brouillette, Heather C. Foil, Annadora J. Bruce-Keller, Hongmei Han, William D. Johnson, Jeffrey N. Keller

**Affiliations:** ^1^Department of Psychology, Louisiana State University (LSU)Baton Rouge, LA, USA; ^2^Institute for Dementia Research and Prevention, Pennington Biomedical Research Center/LSUBaton Rouge, LA, USA

**Keywords:** longitudinal, cognitive decline, falls, gait, older adults

## Abstract

Gait abnormalities are linked to cognitive decline and an increased fall risk within older adults. The present study addressed gaps from cross-sectional studies in the literature by longitudinally examining the interplay between temporal and spatial aspects of gait, cognitive function, age, and lower-extremity strength in elderly “fallers” and “non-fallers”. Gait characteristics, neuropsychological and physical test performance were examined at two time points spaced a year apart in cognitively intact individuals aged 60 and older (*N* = 416). Mixed-model repeated-measure ANCOVAs examined temporal (step time) and spatial (stride length) gait characteristics during a simple and cognitive-load walking task in fallers as compared to non-fallers. Fallers consistently demonstrated significant alterations in spatial, but not temporal, aspects of gait as compared to non-fallers during both walking tasks. Step time became slower as stride length shortened amongst all participants during the dual task. Shorter strides and slower step times during the dual task were both predicted by worse executive attention/processing speed performance. In summary, divided attention significantly impacts spatial aspects of gait in “fallers”, suggesting stride length changes may precede declines in other neuropsychological and gait characteristics, thereby selectively increasing fall risk. Our results indicate that multimodal intervention approaches that integrate physical and cognitive remediation strategies may increase the effectiveness of fall risk interventions.

## Introduction

Falling is the leading cause of both fatal and non-fatal injuries among older adults with serious psychological, physical, and financial implications (Centers for Disease Control and Prevention, 2014). It is estimated that approximately one-third of older adults aged 65 or above fall each year (Centers for Disease Control and Prevention, 2014). Despite these large individual and societal costs, falling within older adults has received relatively little attention in the literature. Relevantly, individuals with cognitive impairment or dementia are at increased risk for falls, and there is an increasing awareness by groups like the American Geriatrics Society/British Geriatrics Society Clinical Practice to include cognitive status in determining fall risk and designing fall prevention interventions for a patient (Kenny et al., 2011). The importance of understanding the interplay between gait and cognition is heightened by recent research that indicates that gait changes, in particular gait changes during different levels of cognitive load, may be related to dementia risk and frontal lobe dysfunction that precedes the development of dementia by decades (Verghese et al., 2006, 2007, 2013, 2014; Hausdorff and Buchman, 2013; Albers et al., 2015). Taken together, these data highlight the public health importance of fall risk research designed to understand the complex interplay between cognition and gait in the elderly.

Real world environments are typically complex and filled with both external and internal distractions. Negotiating real world environments while walking requires the ability to inhibit irrelevant information and sustain attention for general navigation as well as avoiding obstacles and danger (e.g., crossing the street; Neider et al., 2011). An increasing number of studies have identified that monitoring temporal and spatial changes in gait in response to increasing cognitive load (dual task conditions) serves as a useful proxy for everyday walking and the challenges it represents for older adults who may be at a greater risk of falling (e.g., Springer et al., 2006; Allali et al., 2007; Holtzer et al., 2007; Sheridan and Hausdorff, 2007; Hausdorff et al., 2008; Yogev et al., 2008; Herman et al., 2010; MacAulay et al., 2014).

Several studies have found that the specific cognitive processes of executive function and attention are predictive of falls within both clinical and non-clinical elderly populations (Hausdorff et al., 2001; Verghese et al., 2002; Springer et al., 2006; Holtzer et al., 2007; Herman et al., 2010; Buracchio et al., 2011). It is currently not clear how executive function and attention in “fallers” interact with both spatial (e.g., stride length) and temporal (e.g., step time) aspects of gait during periods of increased cognitive load. The present study contributes to the current literature by investigating in fallers and non-fallers the interplay between specific cognitive domains and both spatial and temporal gait characteristics of gait, in a longitudinal manner.

## Methods

### Participants

Research participants are part of an on-going longitudinal study (Louisiana Aging Brain Study: LABrainS) that investigates the effects of aging upon cognitive processes and daily living functioning in relatively healthy older adults (age: ≥ 60 years). LABrainS is an open enrollment longitudinal study that has been following participants since 2009 (overall retention rate of 87%). Participants are recruited throughout Louisiana using traditional media sources (e.g., newspaper ads and television and newspaper press) as well regular community outreach efforts of the Institute for Dementia Research and Prevention (IDRP). Telephone screening procedures are used. Exclusion criteria for LABrainS includes: a Geriatric Depression Scale score ≥ 6 (15 item version; Sheikh and Yesavage, 1986), a history of neurological or untreated health conditions (e.g., cerebrovascular disease, Parkinson’s disease, and/or a traumatic brain injury, etc.) that might cause cognitive impairment. The present study investigated data collected between 2012 and 2013. Only participants with complete data sets for the primary variables of interest (gait, neuropsychological, and fall data) were selected for this study (*N* = 416). All participants were relatively healthy and cognitively intact (Mini-Mental Status Exam scores: *MMSE* = 29.23; Folstein et al., 1975). The sample was primarily Caucasian (91.6%, 4% African American, and 4.4% other). There were a higher proportion of female participants (67.5% female vs. 32.5% male). All participants had normal or corrected vision. Oral and written informed consent was obtained from participants at each clinic visit and the study was approved by the Pennington Biomedical Institutional Review Board and Ethics Committee.

### Measures

Fall history was collected at the 2013 visit. As research suggests that individuals’ conceptions of falling may differ, a structured clinical interview was used to obtain participants’ fall history for the past year. Clinical interview of falls has demonstrated acceptable psychometric properties within non-demented individuals (Holtzer et al., 2007). Consistent with recommended research definitions of falls (Lamb et al., 2005), falls were defined as times that individuals unexpectedly lost their balance and unintentionally came unto rest on the ground, floor or other object; events in which participants were able to regain their balance did not count as a fall (e.g., tripping but catching oneself before falling onto the floor). Individuals were categorized as “fallers” if they had reported a fall within the past 12 months.

The Short Physical Performance Battery (SPPB; Guralnik et al., 1994), a reliable and validated measure of lower extremity strength, was used to assess balance, normal walking speed, and sit-to-stand time^28^. The SPPB three timed tests were summed to create a total SPPB score (maximum score of twelve) for each year.

The GAITRite® system (CIR Systems, Inc., Sparta, NJ) is an electronic-carpet walkway that provides valid and reliable measurement of components that make up an individual’s walking gait (Bilney et al., 2003). Stride length (the line of progression between two consecutive footprints of the same foot in centimeters) and step time (the time elapsed from first contact of one foot to the opposite foot’s first contact in seconds) served as gait measures. Average stride length and step time scores were respectively created for the simple task and dual task conditions at each year.

The MMSE assessed global cognitive functioning. In order to assess whether executive attention/processing speed predicted factors related to falling, an executive attention/processing speed composite score was created based on a factor analysis that confirmed previous research that has found that the Wechsler’s Adult Intelligence Scale-Revised Digit Symbol Subtest (Wechsler, 1987) and the Trails Making Test: Parts A and B (TMT-A, B; Reitan and Wolfson, 1993) load together on an executive attention/processing speed factor (Holtzer et al., 2007). TMT scores were reversed scored before the regressed factor score was created. The three-item scale demonstrated acceptable reliability (*α* = 0.65).

### Study Procedures

The study’s procedures were conducted by well trained, certified research assistants. Each year participants first underwent informed consent followed by neuropsychological testing and the SPPB in a private testing suite. Subsequently, the GAITRite system was administered in an adjacent well-lit hallway for a total of four trials (two per condition with simple task trials always being administered before the dual task trials). For the simple task and dual task trials, participants were instructed to walk across the walkway “using their normal everyday walking speed”. Participants were additionally instructed to spell a word backwards aloud as they walked during the dual task. All words were five letters in length (for word list, see GAITRite manual).

### Analyses

Preliminary analyses used ANOVAs and a Chi-square test to examine for potential differences in demographic variables. Brown–Forsythe test statistic was used to adjust degrees of freedom when assumptions of homogeneity were violated. Males were significantly older, taller and more educated than females, *p*’s < 0.01. Given these differences, subsequent analyses entered sex, age, and height as time invariant covariates (measured at time one). Recommended procedures of centering covariates in Repeated Measures ANCOVA were followed (Thomas et al., 2009), such procedures allow for more sensitive detection of the main effect of task condition while still assessing the interactions between the dependent variables and the covariates. Levene’s statistic initially indicated that assumptions of homogeneity of variance were violated for the gait measures. Visual inspection via box plots revealed eight extreme outliers (>2.5 SDs). Once these outliers were removed assumptions of normality were met and the re-ran analyses results were stable (i.e., significance remained the same whether or not the outliers were included in the dataset). Different ns in conditions are reported and are due to: technical/administrative errors and participant timing constraints. List wise deletion was used to handle missing data.

Mixed-design repeated measure ANCOVAs respectively examined group differences in gait parameters during the simple task and dual task conditions at both time points. Two-sets of ANCOVA analyses were conducted to respectively assess the effects of the covariates (demographic and risk factors) on gait measures. The next set of analysis examined the interrelationships between age, attention, and gait measures during the dual task at 2013 using partial correlations to control for the effect of sex, age, and height on gait measures. Two-tailed tests were used to compute all *p*-values.

## Results

### Demographic and Individual Difference Variables

Table 1 presents the results for ANOVAs with the demographic and individual difference variables grouped by fallers and non-fallers. Groups did not significantly differ in age, education or height, *p*’s > 0.10. Chi-square tests found no association between sex and falling, ${\text{χ}}_{\left(\text{1}\right)}^{\text{2}}$ = 0.05, *p* = 0.82. A trend level difference in SPPB scores were found between fallers and non-fallers only at 2013, *p* = 0.10. No significant differences in standardized composite scores for executive attention/processing speed between fallers (*M* = −0.01, *SD* = 0.88) and non-fallers (*M* = 0.08, *SD* = 0.99) was found, *F*_(1,412)_ = 0.54, *p* = 0.46.

**Table 1:** **Group differences in demographic factors, cognitive functioning, and physical individual difference variables**.

Individual difference variables: *M (SD)*	Non-fallers (*n* = 312)	Fallers (*n* = 81)	*p*-values ≤
Age	70.13 (6.62)	69.90 (6.81)	0.78
Percentage of sample	81.40	18.60	na
Years of education	16.10 (2.50)	16.37 (2.28)	0.50
2012 MMSE	29.25 (1.02)	29.47 (0.87)	0.16
2013 MMSE	29.28 (1.12)	29.22 (1.26)	0.66
Height in centimeters	165.40 (9.61)	164.75 (7.66)	0.59
2012 SPPB	11.02 (1.25)	11.04 (1.40)	0.88
2013 SPPB	11.08 (1.19)	10.82 (1.48)	0.10^†^

*Notes: Mean (M) and Standard Deviation (SD); Mini Mental State Exam (MMSE); SPPB (SPPB); ^†^p < 0.10*.

### Gait Differences by Condition and Group

**Figure 1:**
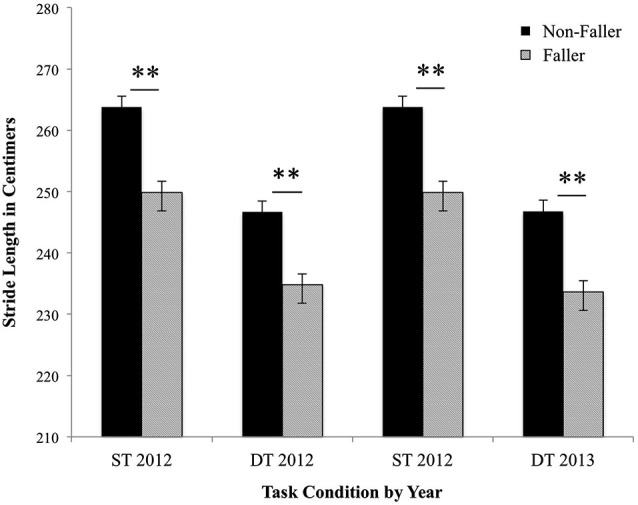
**Fallers vs. Non-fallers group means and standard errors for stride length (cm) by condition and year**. The stride length for non-fallers and fallers was analyzed under single task (ST) and dual task (DT) conditions in 2012 and 2013. Results represent the significant main effect of task condition and the significant main effect of group on stride length. Sex, age, and height were entered as covariates, all *p*’s < 0.01. **p < 0.001.

A mixed-design repeated-measure ANCOVA examined Group (Fallers vs. Non-Fallers) × Task Condition (simple task vs. dual task) × Time (2012 vs. 2013) differences in gait stride length; height, age, and sex served as covariates. Figure 1 presents the group means and errors by condition and time. The covariates of sex, height and age were all significantly related to stride length, such that being female, increased age and shorter height were all associated with shorter stride length, *p*’s < 0.001. A main effect of group found that fallers as compared to non-fallers had significantly shorter stride length, *F*_(1,405)_ = 15.80 *p* < 0.001. No between group interactions with time or condition were found, *p*’s > 0.10. A main effect of task condition revealed that stride significantly shortened during the dual task as compared to the simple task within all participants, *F*_(1,405)_ = 41.41, *p* < 0.001. An interaction between age and task suggests that the effect of significantly shorter stride length during the dual task as compared to the simple task increases with age, *F*_(1,405)_ = 15.26, *p* < 0.001. There was no main effect of time, *F*_(1,405)_ = 1.89, *p* = 0.17; nor were there any interactions with group nor condition, *p*’s > 0.10. Time did significantly interact with age, such that as age increased overtime, stride length also decreased, *F*_(1,405)_ = 5.18, *p* = 0.02. Next, analyses examined the effect of executive attention/processing speed and SPPB scores on the previous results once these factors were entered as covariates. Executive attention/processing speed and SPPB scores were both positively associated with stride length, *p*’s < 0.001. Task condition significantly interacted with executive attention/processing speed (*F*_(1,387)_ = 22.11, *p* < 0.001) and SPPB performance, *F*_(1,387)_ = 6.10, *p* < 0.01. Of interest, the main effect of task was not present once the covariates of executive attention/processing speed and SPPB scores were measured, *F*_(1,405)_ = 0.23, *p* = 0.63. Furthermore, the interaction between age and task was no longer significant, *p* = 0.23.

A mixed-design repeated-measure ANCOVA examined Group (Fallers vs. Non-Fallers) × Task Condition (simple task vs. dual task) × Time (2012 vs. 2013) X differences in gait step time; height, age, and sex served as covariates. Table 2 presents the descriptive statistics for step time by group. The covariates of sex, height and age were all significantly related to step time at both years; such that being male, increased age and shorter height associated with slower step times, *p*’s < 0.001. Step time did not significantly differ between fallers as compared to non-fallers, *F*_(1,405)_ = 0.01, *p* = 0.93; nor was there any significant interactions with group, *p*’s > 0.10. There was a main effect of task condition that revealed that step time significantly increased during the dual task as compared to the simple task within all participants, *F*_(1,405)_ = 14.62, *p* < 0.001. There was also a significant interaction between age and task *F*_(1,405)_ = 15.28, *p* < 0.001; these results indicated that as age increased participants’ step time became marginally slower during the dual task as compared to the single task. There was no main effect of time, *F*_(1,405)_, 1.56, *p* = 0.21; nor were there any significant interactions with time, *p*’s > 0.10. The final analyses examined the effect of executive attention/processing speed and SPPB as covariates on the dependent variable, step time. Executive attention/processing speed and SPPB scores had an inverse relationship with step time. Task condition significantly interacted with executive attention/processing speed, *F*_(1,387)_ = 21.36, *p* < 0.001. No interactions between SPPB performance and step time were found, *p*’s < 0.01. Similar to stride length, the interaction between age and task was no longer significant once executive attention/processing speed and SPPB scores were entered as covariates, *p*’s > 0.10.

**Table 2:** **Group means and standard deviations in step time by task condition at each year**.

Step time in seconds *M (SD)*	Non-fallers (*n* = 333)	Fallers (*n* = 77)
2012 Single task	1.08 (0.09)	1.08 (0.08)
2013 Single task	1.07 (0.09)	1.07 (0.09)
2012 Dual task	1.14 (0.13)	1.13 (0.11)
2013 Dual task	1.12 (0.10)	1.12 (0.11)

*Note(s): Means (M) and Standard Deviations (SD)*.

### Relationships Between Age, Attention, and Gait

Table 3 presents the interrelationships between age and executive attention/processing speed with gait measures during the dual task at 2013. Correlational analysis found that greater age associated with poorer executive attention/processing speed performance, decreased stride length, and increased step times during the dual task. Subsequent analyses used partial correlations to adjust for the effect of sex, age, and height on gait measures. Better executive attention/performance speed performance at 2012 predicted both longer stride length and faster step times during the dual task condition at follow-up. Shortened stride length was significantly associated with slower step times during the dual task.

**Table 3:** **Correlations between age, sex, attention, and dual task gait measures**.

*N* = 415	Age	EA/PS	Stride length	Step time
Age	-	-	-	-
EA/PS	-0.55^**^	-	-	-
Stride length	-0.30^**^	0.24^**^	-	-
Step time	0.29^**^	-0.19^**^	−0.28^**^	-

Notes: Sex, age, and height were entered as covariates with gait measures; Executive Attention/Processing Speed: EA/PS; ** p < 0.001.

## Discussion

The present study longitudinally examined the interplay between cognitive function, temporal and spatial aspects of gait, age, and lower-extremity strength in elderly “fallers” and “non-fallers”. We hypothesized that: (1) fallers would have worse neuropsychological test and physical performance than non-fallers; (2) fallers would have a significantly shorter stride and slower step times than non-fallers within both gait conditions; and (3) stride length would be shorter and step time would increase during the dual task as compared to the simple task within both groups. It was also posited that shorter stride length would associate with slower step times during the dual task, and shortened stride length and slower step times would be predicted by worse cognitive performance. It was further expected that increased age would associate with worse attention performance and gait decrements during the dual task gait condition. Our findings below are discussed in the context of each of these hypotheses and assumptions.

Based on the literature, we predicted that fallers would have worse neuropsychological test and physical performance than non-fallers. In this study, fallers as compared to non-fallers did not significantly differ in executive attention/processing speed performance and demonstrated only a trend level difference in worse physical performance at follow-up.

Although we expected fallers to significantly differ in both cognitive and physical performance aspects based on past research, stride length was the only aspect of gait significantly altered in fallers as compared to non-fallers. Such findings are important in light of research that has linked spatial aspects of gait to white matter atrophy, smaller hippocampal volumes and biomarkers of decreased neuronal viability in relatively healthy older adults (Zimmerman et al., 2009; Callisaya et al., 2013).

We hypothesized that fallers would have a significantly shorter stride length and slower step times than non-fallers during both single and dual task conditions. Indeed, fallers consistently demonstrated shorter stride length than non-fallers within both walking task conditions; however, no significant differences in step time were found between fallers and non-fallers. These effects remained even when sex, age, and height were controlled. These findings and others indicate that decreased stride length precedes or provokes changes in older adults’ step times. Healthy older adults have been shown to compensate for cognitive load during dual task walking conditions by increasing both their stride length and stride times in order to promote gait stability (Li et al., 2012). Whereas in patients with dementia who exhibit gait disturbances, shortened stride length gives rise to slower walking speeds (Beauchet et al., 2008). Taken together, “successful” adaptations to cognitive demands that result in greater gait stability appear to involve pairing slower walking speed with longer strides. In this respect, the ability to effectively coordinate stride length with step time may serve as useful heuristics of fall risk.

Consistent with our hypotheses, when gait is analyzed irrespective of fall history, shorter stride length and slower step times were found among all participants during the cognitive-load walking task as compared to the simple walking task. Furthermore, walking speed (as measured by step time) became slower as stride length shortened across all participants as a function of cognitive load during the dual task. Moreover, shorter stride length and slower step time during the dual task were both predicted by worse executive attention/processing speed performance. Furthermore, as expected the respective interactions found with task condition indicated that the negative effect of cognitive load on gait characteristics is moderated by increased age, executive attention/processing speed impairments, and loss of lower physical extremity strength. Of interest, adjusting for differences in executive attention/processing speed and physical performance attenuated the relationship between age and gait abnormalities. Our results suggest that stride length shortens as a function of decrements in attentional processes, which in turn gives rise to slower walking speeds in older adults. Altogether these results indicate that attention decrements significantly impact stride length, and that individuals with an already shorter stride length are at a greater risk of falling when under cognitive load/distraction due to the destabilizing interaction between slower walking speed and shortened stride length.

A potential limitation to this study is that LABrainS participants are generally college educated, predominantly white, with a higher proportion of females than males, which may limit the generalizability of these findings. Another potential is the time frame of one-year. It is possible that the failure to find gait changes overtime may have been limited by the relatively small time frame of one-year. Additionally, while retrospective clinical interview for fall history has demonstrated acceptable reliability within the literature, there is still the possibility that some individuals may have inaccurately reported their falls due to failure to recall memories of these events. In this respect, prospective collection of fall history at multiple time points through out the time period of interest has distinct advantages over retrospective collection at a single time point; however this methodology contains its own set of limitations due to the greater burden placed upon the participants which can result in greater participant bias and missing data. Overall, despite these limitations, our results are important in that stride length was an important and stable individual difference variable between fallers and non-fallers that interacted with dual task attention decrements. Additionally, these results indicate that future studies may need to uniformly assess for the influence of sex, age, and height when investigating gait characteristics.

From a treatment perspective, there is some initial support that cognitive remediation training can improve balance and gait speed in older adults during cognitive distraction walking tasks. However, these beneficial effects were primarily limited to those categorized as “slow” walkers (Smith-Ray et al., 2013). Thus, whether cognitive remediation can improve stride length is uncertain. Interventions that have targeted shortened stride length in individuals with Parkinson’s disease have found that the beneficial effects of stride length training are negated when participants are required to perform a cognitive dual task (Morris et al., 1996). Given the complexity of the factors involved in maintaining gait stability, it appears that taking a multimodal intervention approach that integrates physical and cognitive remediation strategies may increase the effectiveness of fall risk interventions.

Our previous research has found that the magnitude of contribution of cognitive test performance to gait speed variance during dual task walking conditions increases in accord with cognitive decline (MacAulay et al., 2014). Such findings are also consistent with research that has found that gait changes precede observable cognitive impairment and increased fall risk. In this respect, gait characteristics that share certain neural substrates (e.g., superior parietal cortex and dlPFC) with executive function and attention processes may serve as sensitive early indicators of cognitive decline and fall risk (see Sheridan and Hausdorff, 2007). In all, gait is a dynamic process and these results appear to indicate that spatial aspects of gait require further investigation, as stride length changes may precede declines in other neuropsychological and gait characteristics that lead to an increased risk of falling.

## Author Contributions

RKM, AJB and JNK were responsible for the conception and design of the experiments. RKM and JNK were responsible for analyzing the data and writing the paper. RKM, TDA, HCF and RMB performed the experiments. HCF, RMB, HH, and WDJ contributed materials/analysis tools.

## Sponsors’ Role

Funding for this study came from the Hibernia National Bank/Edward G Schlieder Chair and independent community supporters of the Institute for Dementia Research and Prevention. The funders of this manuscript had no role in the study design, data collection and analysis, decision to publish, or preparation of the manuscript.

## Conflict of Interest Statement

The authors declare that the research was conducted in the absence of any commercial or financial relationships that could be construed as a potential conflict of interest.
